# β-Xylosidases: Structural Diversity, Catalytic Mechanism, and Inhibition by Monosaccharides

**DOI:** 10.3390/ijms20225524

**Published:** 2019-11-06

**Authors:** Ali Rohman, Bauke W. Dijkstra, Ni Nyoman Tri Puspaningsih

**Affiliations:** 1 Department of Chemistry, Faculty of Science and Technology, Universitas Airlangga, Surabaya 60115, Indonesia; alirohman@fst.unair.ac.id; 2 Laboratory of Proteomics, Research Center for Bio-Molecule Engineering (BIOME), Universitas Airlangga, Surabaya 60115, Indonesia; 3 Laboratory of Biophysical Chemistry, University of Groningen, 9747 AG Groningen, The Netherlands; b.w.dijkstra@rug.nl

**Keywords:** biomass, hemicellulose, bioethanol, xylanolytic enzyme, hemicellulase, glycoside hydrolase

## Abstract

Xylan, a prominent component of cellulosic biomass, has a high potential for degradation into reducing sugars, and subsequent conversion into bioethanol. This process requires a range of xylanolytic enzymes. Among them, β-xylosidases are crucial, because they hydrolyze more glycosidic bonds than any of the other xylanolytic enzymes. They also enhance the efficiency of the process by degrading xylooligosaccharides, which are potent inhibitors of other hemicellulose-/xylan-converting enzymes. On the other hand, the β-xylosidase itself is also inhibited by monosaccharides that may be generated in high concentrations during the saccharification process. Structurally, β-xylosidases are diverse enzymes with different substrate specificities and enzyme mechanisms. Here, we review the structural diversity and catalytic mechanisms of β-xylosidases, and discuss their inhibition by monosaccharides.

## 1. Introduction

**Figure 1:**
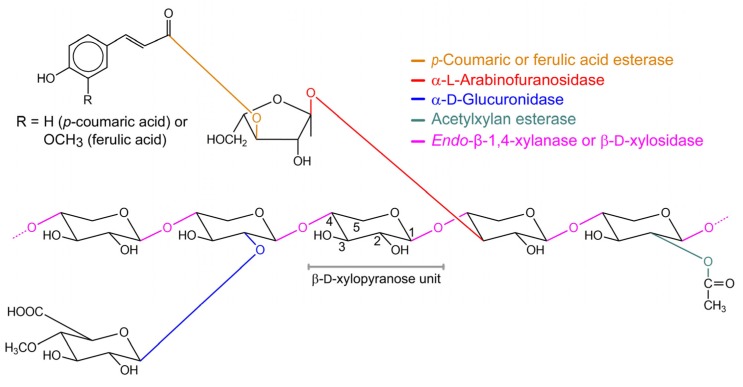
Example of the structure of a plant xylan with the cleavage sites of various xylanolytic enzymes indicated. A β-d-xylopyranose unit with numbered carbon atoms is shown in the middle. Glycosidic bonds and xylanolytic enzymes that hydrolyze them are depicted in the same color [7,9].

Xylan is a prominent component of cellulosic biomass, a heterogeneous complex of carbohydrate polymers (cellulose and hemicellulose) and lignin, a complex polymer of phenylpropane units [1,2,3]. Hemicellulose, including xylan, makes up approximately one-third of the carbohydrate content of common agricultural and forestry waste [1,2,3]. It is among the most inexpensive non-food biomass that is sustainably available in nature in large quantities, and that can be converted into biofuel or other value-added products, such as low-calorie sweeteners, prebiotics, surfactants and various specialty chemicals [1,4,5,6]. Structurally, xylan is a complex heteropolysaccharide with a glycosidic β-(1,4)-linked d-xylose backbone that is frequently substituted with side chains of arabinose, glucuronic acid and other groups. In turn, these side chains may be further esterified with acetic, ferulic, and *p*-coumaric acids (Figure 1). The type and frequency of the side chains and their substituents vary with the source of xylans [2,7,8,9,10,11].

As a complex heteropolysaccharide, full degradation of xylan into its monosaccharide constituents requires the concerted action of various hydrolytic xylan-degrading enzymes with different specificities (Figure 1). These enzymes include α-l-arabinofuranosidase (EC 3.2.1.55), α-d-glucuronidase (EC 3.2.1.139), acetylxylan esterase (EC 3.1.1.72), and *p*-coumaric acid and ferulic acid esterases (EC 3.1.1.73), which release the side chain substituents from the xylan backbone, and *endo*-β-1,4-xylanase (EC 3.2.1.8), which works synergistically with β-xylosidase (EC 3.2.1.37) to break down the xylan backbone. *Endo*-β-1,4-xylanase hydrolyses the internal β-(1,4) linkages of the xylan backbone producing short xylooligosaccharides, while β-xylosidase removes xylose units from the non-reducing termini of these xylooligosaccharides [2,7,8,10]. In nature, xylanolytic enzymes are mainly found in numerous saprophytic microorganisms, such as fungi, actinomycetes and other bacteria, as well as in the rumen biota of higher animals. The microorganisms secrete the enzymes, for example, as a strategy for expanding their versatility to use primary carbon sources [7,11,12,13,14]. 

Xylan-degrading enzymes have found application as environmentally friendly agents in a wide range of industrial processes, such as bleaching of paper pulp, deinking of recycled paper, enhancing the digestibility and nutritional properties of animal feed, degumming of plant fiber sources, manufacturing of beer and wine, clarification of fruit juices and maceration of fruits and vegetables, preparation of high-fiber baked goods, and the extraction of coffee [2,7,15,16]. Furthermore, the enzymes are applied during the saccharification of pretreated agricultural and forestry cellulosic biomass into fermentable sugars [2,15], e.g., for producing biofuel.

In xylan saccharification, β-xylosidase is a crucial enzyme since, of all the xylanolytic enzymes, it cleaves the greatest number of glycosidic bonds. [17,18,19]. In addition, because xylooligosaccharides are potent inhibitors of *endo*-β-1,4-xylanases and cellulases, the activity of β-xylosidase can improve the efficiency of the saccharification process by degrading the xylooligosaccharides and thus alleviating inhibition of those enzymes [7,11,20,21,22,23]. However, most of the characterized β-xylosidases are, to some extent, also inhibited themselves by xylose, arabinose, glucose, and/or other monosaccharides [2,11,24,25,26,27]. This is an important problem, since in industrial cellulosic biomass saccharification, the monosaccharides may accumulate to high enough concentrations to significantly reduce the activity of β-xylosidase, even in simultaneous saccharification and fermentation processes, where monosaccharides are directly consumed by the fermenting organisms [26,27]. This adverse property may severely reduce the efficiency of β-xylosidases in the saccharification process.

In this review, the structural diversity, catalytic mechanisms and inhibition by monosaccharides of β-xylosidases are discussed.

## 2. Structural Diversity of β-xylosidases

β-Xylosidases are a group of structurally diverse enzymes with varying specificities, in line with the diversity of the organisms that produce them and the heterogeneity of their substrates [28]. However, as commonly observed in other glycoside hydrolases (GHs), they hydrolyze the glycosidic bond via one of two routes, either with overall retention or with overall inversion of the anomeric carbon configuration [29]. 

GHs are classified in the Carbohydrate-Active Enzymes database (CAZy; http://www.cazy.org/), which groups the enzymes into families based on their amino acid sequence similarities [30,31]. As there is a direct relationship between amino acid sequence similarity and similarity of folding, the classification also represents the structural features and commonality of the catalytic mechanism of the enzymes. Thus, enzymes in a particular family display highly similar three-dimensional structures and catalytic mechanisms [29,32]. At present, 161 GH families (GH1 to GH161) are represented on the CAZy server. Nevertheless, despite divergent amino acid sequences, several different GH families show significantly similar protein folding and active site architecture. Such GH families are considered to have a common ancestor and, therefore, have been grouped together into a clan [33]. To date, 18 GH clans (GH-A to GH-R) have been assigned in the database. 

A search using the enzyme classification number for β-xylosidase (EC 3.2.1.37) in the CAZy database [31] revealed that enzymes with this number are presently found in 11 different GH families (Table 1). Nevertheless, a further literature examination suggests that 3 families, i.e., GH1, GH54 and GH116, may not contain enzymes with β-xylosidase activity on natural substrates. The enzymes from *Reticulitermes flavipes* (RfBGluc-1; GenPept accession No. ADK12988) [34] and *R. santonensis* De Feytaud (GenPept ADT62000) [35] in GH1, and *Trichoderma koningii* G-39 (TkAbf; GenPept AAA81024) [36] in GH54 were classified as β-xylosidases because they hydrolyze artificial nitrophenyl-β-d-xylopyranoside derivatives. However, to our knowledge there is no evidence that these enzymes are able to release xylose from natural substrates. Similarly, a bifunctional aryl β-glucosidase/β-xylosidase from the hyperthermophilic archaeon *Saccharolobus solfataricus* P2 (formerly *Sulfolobus solfataricus*; SSO1353; GenPept AAK41589) in GH116 is called so based on its activity on aryl β-glucosides and β-xylosides, but the enzyme likely does not hydrolyze xylooligosaccharides [37]. All in all, this suggests that enzymes with β-xylosidase activity on natural substrates currently occur in only 8 GH families in the CAZy database, i.e., in GH families 3, 5, 30, 39, 43, 51, 52 and 120. 

### 2.1. Glycoside Hydrolase Clan A (GH-A)

GH families 1, 5, 30, 39 and 51 are part of clan GH-A, the largest clan in the CAZy database with currently 23 GH families. Enzymes in this clan all have a (β/α)_8_ catalytic domain, also known as triose-phosphate isomerase (TIM) barrel domain [39].

**Figure 2:**
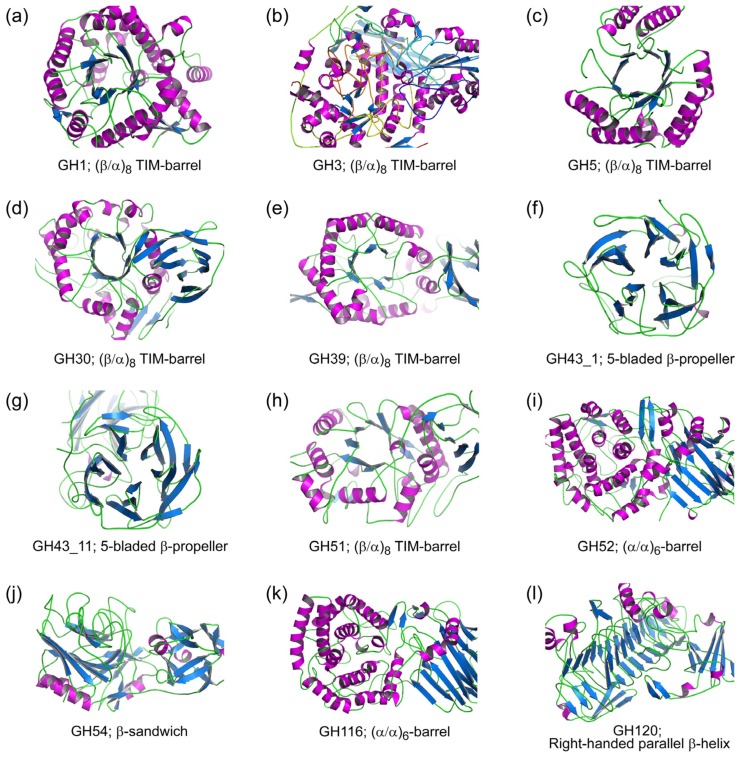
Three-dimensional (3D) structures of β-xylosidases from various GH families. Helix, strand, and loop structures are colored in magenta, blue, and green, respectively. GH family numbers and fold type of their catalytic domains are shown. The structures represented are (**a**) RfBGluc-1 from *Reticulitermes flavipes* (GenPept ADK12988); (**b**) GlyA1 from metagenomic cow rumen fluid (PDB 5K6L); (**c**) PcXyl5 from *Phanerochaete chrysosporium* BKM-F-1767 (GenPept AHL69750) (**d**) PiBGX1 from *Phytophthora infestans* (GenPept AAK19754); (**e**) TsXynB from *Thermoanaerobacterium saccharolyticum* B6A-RI (PDB 1PX8); (**f**) RS223-BX from an uncultured organism (PDB 4MLG); (**g**) GsXynB3 from *Geobacillus stearothermophilus* T-6 (PDB 2EXH); (**h**) AtAraf from *Arabidopsis thaliana* (GenPept AAF19575); (**i**) GT2_24_00240 from *Geobacillus thermoglucosidasius* TM242 (PDB 4C1O); (**j**) TkAbf from *Trichoderma koningii* G-39 (GenPept AAA81024); (**k**) SSO1353 from *Saccharolobus solfataricus* P2 (GenPept AAK41589); and (**l**) TsXylC from *Thermoanaerobacterium saccharolyticum* JW/SL-YS485 (PDB 3VST). The structures of (a), (c), (d), (h), (j), and (k) were modeled using PDB entries 3VIK, 1EQP, 2XWE, 2C8N, 1WD3, and 5BVU, respectively, which belong to the same GH family but do not have β-xylosidase activity. Structure modeling was performed using the Swiss-Model server [46]. Figure 2, Figure 3, and Figure 6 were produced using the program PyMol (The PyMOL Molecular Graphics System, v. 0.99, Schrödinger, LLC, http://www.pymol.org).

Of clan GH-A, structural data for β-xylosidases are currently only available for GH39, i.e., β-xylosidases from *Thermoanaerobacterium saccharolyticum* B6A-RI (TsXynB; Protein Data Bank code 1px8; Figure 2e) [40], *Geobacillus stearothermophilus* T-6 (GsXynB1; PDB 2BS9) [41] and *Caulobacter crescentus* NA1000 (CcXynB2; PDB 4EKJ) [42]. These enzymes fold into a three-domain structure, consisting of an N-terminal (β/α)_8_-barrel catalytic domain, sequentially followed by a β-sandwich and an α-helical accessory domain. Their structures are very similar. Superposition of the structures of isolated proteins gave an overall root mean squared deviation (RMSD) of 1.6 Å for 462 amino acid residues. However, while CcXynB2 exists as a monomer in solution [42], TsXynB and GsXynB1 are present as tetramers [40,41]. The absence of a short amino acid sequence at the C-terminus of CcXynB2, compared to the other two enzymes, has been suggested to prevent the formation of a stable tetramer [42]. Additionally, it has been proposed that subtle structural differences in the accessory domains of these β-xylosidases slightly alter their overall structure and the accessibility of their catalytic region [42].

In the absence of structural data for β-xylosidases from families GH1, GH5, GH30, and GH51 we generated homology models to compare the 3D structures of β-xylosidases from the different families of the GH-A clan. Models were built of the β-glucosidase/β-xylosidase RfBGluc-1 (GH1; Figure 2a) [34], a β-xylosidase from *Phanerochaete chrysosporium* BKM-F-1767 (PcXyl5; GenPept AHL69750; GH5; Figure 2c) [43], a β-glucosidase/β-xylosidase from *Phytophthora infestans* (PiBGX1; GenPept AAK19754; GH30; Figure 2d) [44], and an α-l-arabinofuranosidase/β-xylosidase from *Arabidopsis thaliana* (AtAraf; GenPept AAF19575; GH51; Figure 2h) [45], using 3D structures of their nearest homologs as templates. All resulting models display a (β/α)_8_-barrel catalytic domain that is highly similar to the catalytic domain of GH39 β-xylosidases (e.g., Figure 2e) and that shows that the catalytic residues of GH39 are present at the equivalent positions in the GH1, GH5, GH30, and GH51 β-xylosidase families. A multiple structural alignment of the catalytic domains of these models and GH39 β-xylosidases gave an overall RMSD of 3.4 Å for 168 amino acid residues, with PcXyl being the most divergent from the other structures. In contrast, the structures of their accessory domains varied with the family. The accessory domains of GH39 β-xylosidases are absent in RfBGluc-1 and PcXyl5, but they are retained at a comparable position in PiBGX1 and AtAraf albeit with some modifications. The major differences are observed for the third domain, in which the GH39 α-helical domain is replaced by a β-sheet and a loop structure in PiBGX1 and AtAraf, respectively.

### 2.2. Glycoside Hydrolase Family 3 (GH3)

While GH families 1, 30, 39 and 51 are part of clan GH-A, other β-xylosidases belong to other families that are not part of this clan. GH3 is one of the largest and most diverse GH families in the CAZy database [28,47]. It contains more than 23400 entries with various enzyme activities, including β-xylosidase, β-glucosidase, β-glucosylceramidase, β-N-acetylhexosaminidase, and α-l-arabinofuranosidase activities. A number of GH3 enzymes are reported to be bi/multifunctional, particularly toward synthetic substrates.

Enzymes in family GH3 vary considerably in the lengths of their peptide chains [48,49] and, consequently, in the number of tertiary structure domains [48,50]. The basic structure of GH3 members is a single (β/α)_8_ TIM-barrel domain [48,50], similar to the domain that is observed in clan GH-A. In most members, the domain is followed by an (α/β)-sandwich domain that varies in size [48], e.g., (α/β)_6_ in *Kluyveromyces marxianus* NBRC1777 β-glucosidase [51], (α/β)_5_ in *Thermotoga neapolitana* β-glucosidase [49], or even only an αβα motif in *Bacillus subtilis* 168 β-N-acetylglucosaminidases [52]. Sometimes the order of the domains in the primary structure is reversed [48]. Although these two domains are generally sufficient to organize the active site of GH3 enzymes, frequently GH3 members are extended with a fibronectin type III (FnIII) domain of unknown function at the C-terminus of the (α/β)-sandwich domain [48,49]. Moreover, in some GH3 members, the (α/β)-sandwich domain is interrupted by a PA14 domain. This domain appeared to be important for the substrate specificity of the *Kluyveromyces marxianus* NBRC1777 β-glucosidase [51].

A total of 103 enzymes with β-xylosidase annotation are currently found in GH3, making it the largest β-xylosidase-containing GH family. A protein domain search using the program InterProScan 5 [53] revealed that the majority of the GH3 β-xylosidases are composed of three domains (TIM-barrel, (α/β)-sandwich and FnIII). However, a bifunctional β-xylosidase/β-glucosidase from *Erwinia chrysanthemi* D1 (EcBgxA; GenPept AAA80156) [54] has two domains (TIM-barrel and (α/β)-sandwich) and a β-xylosidase from an environmental sample (G06-24; GenPept ACY24766) [55] has four domains (TIM-barrel, (α/β)-sandwich, FnIII, and PA14). A phylogenetic analysis clustered these two enzymes divergently from the other GH3 β-xylosidases [56].

3D Structures of GH3 β-xylosidases are available for a β-xylosidase from the fungus *Trichoderma reesei* RutC-30 (TrBxl1; PDB 5A7M; GenPept CAA93248) [57] and a β-glucosidase/β-xylosidase from metagenomic cow rumen fluid (GlyA1; PDB 5K6L; Figure 2b) [58]. Both structures have a (β/α)_8_ TIM-barrel, a (α/β)_6_-sandwich, and a FnIII domain, but at different positions in the primary structure. As observed for the majority of GH3 structures [48], TrBxl1 has its TIM-barrel domain at the N-terminus, followed sequentially by the (α/β)-sandwich and FnIII domains. This order is reversed in GlyA1, where the (α/β)-sandwich domain is at the N-terminus, followed by the FnIII and TIM-barrel domains. In addition, GlyA1 has an additional domain with unknown structure at its C-terminus [58]. Despite this, the 3D structures of TrBxl1 and GlyA1 are conserved, with the TIM-barrel and (α/β)-sandwich domains, as well as the catalytic residues superimposing reasonably well when the domains are structurally aligned.

### 2.3. Glycoside Hydrolase Family 43 (GH43)

GH43 is the second largest β-xylosidase-containing GH family with currently 96 members annotated as β-xylosidase. In addition to β-xylosidases, this family also contains enzymes with (putative) α-l-arabinofuranosidase, arabinanase, xylanase, galactan 1,3-β-galactosidase, α-1,2-l-arabinofuranosidase, *exo*-α-1,5-l-arabinofuranosidase, *exo*-α-1,5-l-arabinanase, or β-1,3-xylosidase activities. As observed for the GH3 members, several enzymes in this family are bi/multifunctional.

Together with GH62, GH43 is grouped into clan GH-F in the CAZy database with a structural characteristic of a 5-bladed β-propeller catalytic domain [59]. Some of its members contain only this single catalytic domain, and, based on their domain architecture, were classified as type I [60]. In other members, the catalytic domain is extended with a family 6 carbohydrate-binding module (type II), or a unique β-sandwich domain that is designated as X19 [61] (type III), or contain an even more complex domain composition and organization (type IV). The extensions are commonly fused at the C-terminus of the catalytic domain [60,62], although in a β-xylosidase from *G. thermoleovorans* IT-08 (GbtXyl43B), for example, the extension is at the N-terminus [63]. Thus, GH43 contains enzymes that vary both in the lengths of their primary structure and in their number of structure domains.

For detailed characterization, enzymes in GH43 have been divided into 37 subfamilies, GH41_1 to GH43_37 [61]. In this classification, β-xylosidases are currently found in 16 different subfamilies, with the majority belonging to subfamilies GH43_1 and GH43_11. Two GH43_1 β-xylosidase crystal structures are currently present in the PDB database, i.e., from an uncultured organism (RS223-BX; PDB 4MLG; Figure 2f) [64] and from a compost metagenome (CoXyl43; PDB 5GLK) [65]. The most structurally characterized β-xylosidases are from GH43_11, with crystal structures available of seven different β-xylosidases, i.e., the β-xylosidases from *B. subtilis* (PDB 1YIF) (Patskovsky et al., unpublished work), *Clostridium acetobutylicum* ATCC 824 (CaXyl43_11; PDB 1YI7) (Teplyakov et al., unpublished work), *B. halodurans* (PDB 1YRZ) (Fedorov et al., unpublished work), *G. stearothermophilus* T-6 (PDB 2EXH; Figure 2g) [66], *Selenomonas ruminantium* GA192 (PDB 3C2U) [67], *B. pumilus* IPO (PDB 5ZQJ) [68], and *Bacillus* sp. HJ14 (PDB 6IFE) [69]. Additionally, GH43 β-xylosidase 3D structures are also found in subfamilies GH43_12 and GH43_26, i.e., the β-xylosidases from *G. thermoleovorans* IT-08 (PDB 5Z5D) [70] and *C. acetobutylicum* ATCC 824 (CaXyl43_26; PDB 3K1U) (Osipiuk et al., unpublished work), respectively.

While β-xylosidases from GH43_1 and GH43_26 have only a single 5-bladed β-propeller catalytic domain [64,65] and belong to type I GH43 [60], those from GH43_11 and GH43_12 possess an additional X19 domain at the C-terminus [66,67,68,70] and belong to type III GH43 [60]. Although the architecture of the 5-bladed β-propeller is highly conserved among the GH43 β-xylosidases [64], structural superposition of the type I and type III catalytic domains gave a high RMSD. This is because the catalytic domains of the type I enzymes have several significantly longer loops than those of type III. The single catalytic domain of the type I GH43 β-xylosidases is sufficient for activity, but the enzymes are strongly activated by divalent metal ions, particularly calcium. Indeed, those metal-containing enzymes contain a metal-binding site close to the enzymes’ active site [64,65]. In contrast, the type III GH43 β-xylosidases have no such metal-binding site [66,67,68,70]. The X19 domain, which is only found in a subset of GH43 subfamilies [61], appeared to be crucial for catalytic activity of the type III GH43 β-xylosidases, since removing this domain abolished the activity of the GH43_11 β-xylosidases from *Thermobifida fusca* YX [71] and *Enterobacter* sp. [72]. In fact, a loop from the X19 domain contributes a Phe residue to the active site of the type III β-xylosidases [66,67,68,70], which is spatially conserved among all GH43 β-xylosidase structures. Only in CaXyl43_26 this Phe is missing. Unfortunately, no biochemical evidence is available on the enzyme’s substrate preferences and catalytic activity, but given that all other enzymes in GH43_26 are α-l-arabinofuranosidases [61], some doubt that CaXyl43_26 is a genuine β-xylosidase seems justified. These observations suggest that although GH43 β-xylosidases adopt different overall folds, the enzymes have a common active site organization and use a conserved Phe to interact with the substrate in subsite −1 (see below; Figure 4b).

### 2.4. Glycoside Hydrolase Family 52 (GH52)

Currently, GH52 contains 112 entries, of which 11 enzymes are annotated as β-xylosidases. These β-xylosidases have comparable amino acid sequence lengths of about 700 amino acid residues, with the exception of a β-xylosidase from *G. stearothermophilus* 236 (GsXylA, GenPept AAA50863), which is composed of only 618 amino acid residues. The GH52 β-xylosidases are very similar to each other with amino acid sequence identities of around 41%–90%. In this family, crystal structures are available for β-xylosidases from *Parageobacillus thermoglucosidasius* TM242 (GT2_24_00240; PDB 4C1O; Figure 2i) [73] and *G. stearothermophilus* T-6 (Xyn52B2; PDB 4RHH) (Dann et al., unpublished work). With a sequence identity of 86%, the two proteins fold into almost the same structures; they display two distinct domains, an N-terminal β-sandwich domain and a C-terminal (α/α)_6_-barrel domain. The catalytic residues of the GH52 enzymes, which are Glu-357 and Asp-517 in GT2_24_00240 [73], are located in the (α/α)_6_-barrel domain. Protein homology modeling based on the structure of GT2_24_00240 suggested that the domains are conserved among the GH52 β-xylosidases, except for the C-terminal domain of GsXylA. Compared to other GH52 β-xylosidases, this latter domain lacks five α-helices of the C-terminal domain, such that it displays an open half-barrel structure.

### 2.5. Glycoside Hydrolase Family 54 (GH54)

Most of the characterized enzymes in GH54 are annotated as α-l-arabinofuranosidases. However, two sequences in this family are annotated as β-xylosidase. TkAbf from *T. koningii* G-39 was characterized as a bifunctional α-l-arabinofuranosidase/β-xylosidase due to its activity on synthetic nitrophenyl derivatives of α-l-arabinofuranoside and β-d-xylopyranoside with comparable *k_cat_*/*K_m_* values [36]. A three-dimensional structure of a GH54 enzyme is currently only available for an α-l-arabinofuranosidase from *A. kawachii* IFO4308 (AkAbfB; PDB 1WD3) [38]. The primary structures of TkAbf (500 residues) and AkAbfB (499 residues) are very similar with an amino acid sequence identity of 73%. Therefore, the three-dimensional structure of TkAbf was predicted by homology modeling using the crystal structure of AkAbfB as a template. The predicted model (Figure 2j) consists of two domains that correspond to the N-terminal catalytic domain and the C-terminal arabinose-binding domain of the AkAbfB structure [38]. The catalytic domain folds into a β-sandwich similar to that of clan GH-B enzymes, while the arabinose-binding domain has a β-trefoil structure that belongs to the family 42 carbohydrate-binding module [28,38].

### 2.6. Glycoside Hydrolase Family 116 (GH116)

In GH116, SSO1353 is the only enzyme that exhibits β-xylosidase activity. As mentioned above, this enzyme does not hydrolyze xylooligosaccharides, but it is active on artificial substrates such as *p*-nitrophenyl- and methylumbelliferyl-linked β-d-xylopyranosides [37]. Currently, a three-dimensional structure of a GH116 member is only available for the β-glucosidase from the thermophilic bacterium *T. xylanolyticum* LX-11 (TxGH116; PDB 5BVU). This protein folds into an N-terminal β-sandwich domain and a C-terminal (α/α)_6_ solenoid catalytic domain [74]. The primary structure similarity of SSO1353 and TxGH116 is rather low with an amino acid sequence identity of only ~20%. However, homology modeling of SSO1353 based on the structure of TxGH116 using the Swiss-Model server [46] produced a relatively good quality model with a Global Model Quality Estimation (GMQE) value of 0.63 (on a scale of 0–1). As expected, the model displays a two-domain fold, i.e., an N-terminal β-sandwich domain and a C-terminal (α/α)_6_-barrel domain (Figure 2k), very much like the domain organization of the GH52 proteins (see above). Importantly, the modeling placed the catalytic nucleophile and acid/base residues of SSO1353 (Glu-335 and Asp-462, respectively) [37] at about the same positions as those of the GH52 β-xylosidase GT2_24_00240 (Glu-357 and Asp-517, respectively) [73]. In view of this structural similarity and the conservation of the catalytic residues, GH families 52 and 116 were recently grouped into clan GH-O [74].

### 2.7. Glycoside Hydrolase Family 120 (GH120)

Of the 176 sequences that are currently available in the CAZy database for the GH120 family, two enzymes were characterized and identified as β-xylosidases, i.e., enzymes from *Thermoanaerobacterium saccharolyticum* JW/SL-YS485 (TsXylC; GenPept ABM68042) [75] and *Bifidobacterium adolescentis* LMG10502 (BaXylB; GenPept BAF39080) [56]. While TsXylC was shown to be active on xylobiose and xylotriose [75], BaXylB prefers xylotriose or longer xylooligosaccharides as its substrate [56]. The three-dimensional structure of TsXylC has been reported to fold into a core domain of a right-handed parallel β-helix, a common fold observed in several GHs, polysaccharide lyases, and carbohydrate esterases. This core domain is intervened by an Ig-like β-sandwich domain (PDB 3VST, Figure 2l). Both domains are important to organize the active site of the enzyme [25]. BaXylB shares 47% amino acid sequence identity with TsXylC. A homology model of BaXylB based on the structure of TsXylC suggested that the active site residues and their positions are conserved in the enzymes, except for Trp-362 in BaXylB, which is a histidine (His-352) in TsXylC.

## 3. Active Site of β-Xylosidases

**Figure 3:**
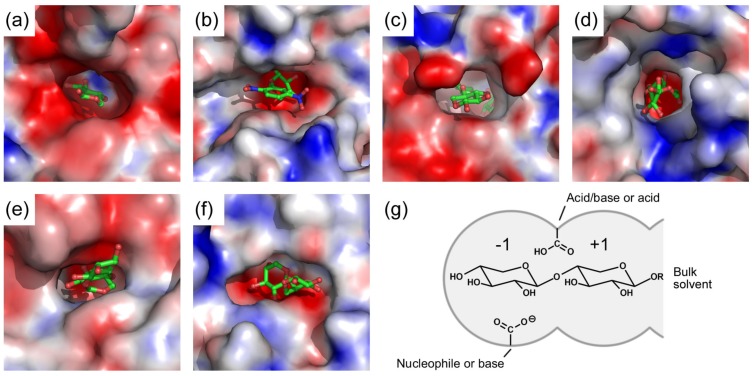
β-Xylosidase active site. Molecular surface drawing of active sites of β-xylosidases colored according to their electrostatic potential (negative, red; neutral, white; positive, blue). Complexed ligands are depicted in ball and stick representation with carbon atoms in green. The active sites are of (**a**) GlyA1 from metagenomic cow rumen fluid in complex with xylose (PDB 5K6N; GH3); (**b**) GsXynB1 from *Geobacillus stearothermophilus* T-6 in complex with 2,5-dinitrophenyl-β-d-xyloside (PDB 2BFG; GH39); (**c**) CoXyl43 from a compost metagenome in complex with xylose and xylobiose (PDB 5GLN; GH43_1); (**d**) GsXynB3 from *G. stearothermophilus* T-6 in complex with xylobiose (PDB 2EXJ; GH43_11); (**e**) GT2_24_00240 from *Parageobacillus thermoglucosidasius* TM242 in complex with xylobiose (PDB 4C1P; GH52); and (**f**) TsXylC from *Thermoanaerobacterium saccharolyticum* JW/SL-YS485 in complex with xylobiose (PDB 3VSU; GH120). The electrostatic potential was calculated using the APBS (Adaptive Poisson–Boltzmann Solver) implemented in the program PyMol [76]. (**g**) Generalized schematic diagram of a β-xylosidase active site with a ligand bound at subsites –1 and +1. Catalytic residues (see below) are represented by carboxylate groups and their catalytic roles are indicated. The exact positions of the catalytic residues vary with enzymes (see Figure 6).

Despite the diversity of their three-dimensional folds, all structurally characterized β-xylosidases display a typical pocket-shaped active site (Figure 3) that is very suitable for *exo*-acting enzymes [29]. The pocket is negatively charged due to the presence of several acidic residues, but contains also hydrophobic patches of aromatic residues (Figure 3a–f). It has only a single route for substrates to enter and products to exit. The active site pocket can be virtually divided into two subsites with each of them able to accommodate a monosaccharide residue (Figure 3g). One subsite is buried, and, in several enzyme-xylobiose complexes (e.g., PDBs 2EXJ, 4C1P and 3VSU), interacts with the –1 non-reducing-end xylose (subsite –1), while the other is more open and binds the +1 xylose (subsite +1). Substrates with more than two xylose residues must have the additional residues beyond +1 exposed in the bulk solvent [25,67]. The active site architecture seems to be both necessary and sufficient for β-xylosidase activity of the enzymes. 

**Figure 4:**
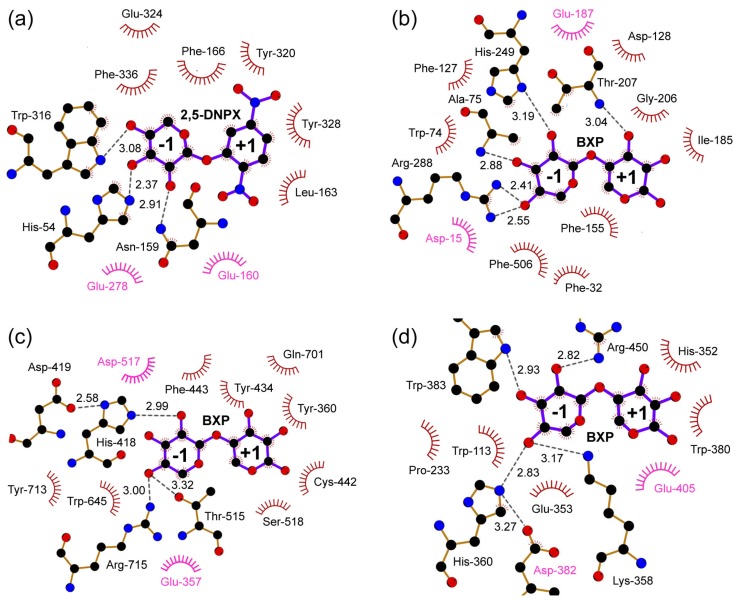
Interactions between active site residues of β-xylosidases and their ligands. The ligands 2,5-DNPX (2,5-dinitrophenyl-β-d-xyloside) and BXP (β-d-xylobiopyranose) are represented with purple bonds and their binding subsites -1 and +1 are indicated. Catalytic residues are labeled in magenta. Hydrogen bonds are shown as dashed lines and their distances are indicated in Å, while hydrophobic interactions are rendered with arcs. The active sites are of (**a**) GsXynB1 (PDB 2bfg); (**b**) GsXynB3 (PDB 2exj); (**c**) GT2_24_00240 (PDB 4c1p); and (**d**) TsXylC (PDB 3vsu), which represent β-xylosidases from GH families 39, 43, 52, and 120, respectively (see caption of Figure 3 for further details of the enzymes). Interaction analysis and figure preparation were performed using LigPlot^+^ [77].

Furthermore, comparison of active site structures of several β-xylosidase-ligand complexes suggests that there are similar interactions between the enzymes and their ligands (Figure 4). The ligand in subsite –1 is strongly bound to the enzyme by a large number of hydrogen bonds and a few hydrophobic stacking interactions. In contrast, the ligand substrate in subsite +1 interacts less strongly with the enzyme with less hydrogen bonds but more hydrophobic stacking interactions.

## 4. Catalytic Mechanism of β-Xylosidases

**Figure 5:**
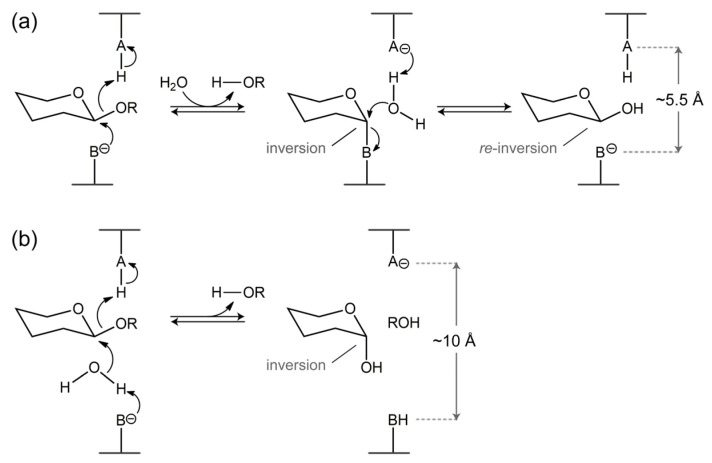
The two common types of catalysis by glycoside hydrolases as adapted from Davies and Henrissat [29]. (**a**) The retaining mechanism. The nucleophile and the general acid/base are represented as B^-^ and AH, respectively. (**b**) The inverting mechanism. The general base and the general acid are represented as B^-^ and AH, respectively. The typical distances of the catalytic residues in both mechanisms are indicated in Å. In most GHs, A and B are either Asp or Glu. See main text for further details.

With respect to their catalytic mechanism most GHs can generally be classified into retaining and inverting enzymes [29,78]. The retaining GHs hydrolyze their substrates with overall retention of the stereochemistry of the anomeric carbon atom of the hydrolyzed glycosidic bond, while the inverting GHs yield a product with an inverted stereochemistry of the anomeric carbon atom [29,78]. In both mechanisms, the enzymes rely on two catalytic carboxylate groups that function as a nucleophile and a general acid/base in the retaining enzymes, or as a general base and a general acid in the inverting enzymes, respectively (Figure 5).

The retaining enzymes use a two-step double-displacement mechanism, in which enzyme glycosylation is followed by deglycosylation. In the glycosylation step, the nucleophile attacks the anomeric carbon to form a glycosyl–enzyme intermediate with the inverted configuration at the anomeric carbon. Concomitantly, the (protonated) acid/base residue transfers its proton to the glycosidic oxygen atom, to cleave the scissile glycosidic bond. Departure of the aglycone creates space allowing a catalytic water molecule to come closer to the anomeric center. In the deglycosylation step, the incoming catalytic water molecule, which is activated by the now negatively charged acid/base, attacks the anomeric carbon to release the glycone product from the intermediate. The attack re-inverts the inverted configuration of the anomeric carbon and hence the released glycone has the same stereochemistry as it had in the substrate. In contrast, the inverting enzymes follow a single-displacement mechanism to hydrolyze the glycosidic bond. A catalytic water molecule, which is deprotonated by the general base, does a nucleophilic attack on the anomeric carbon in concert with the general acid protonating the glycosidic oxygen. This cleaves the scissile glycosidic bond and frees the glycone with the inverted stereochemistry of its anomeric carbon.

**Figure 6:**
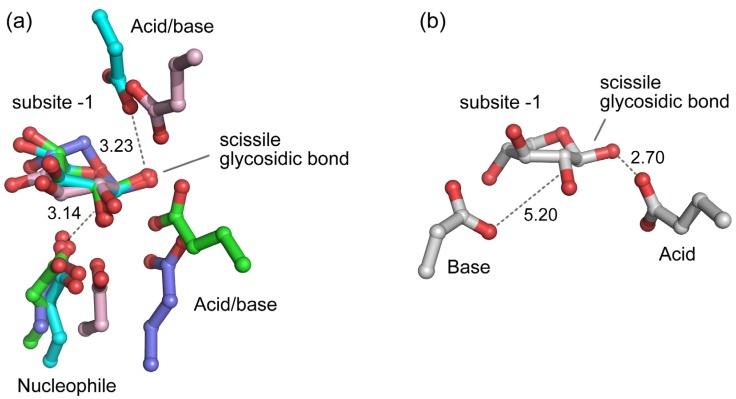
Positions of the catalytic residues relative to the xylosyl moiety bound in subsite -1 of the active sites of (**a**) retaining and (**b**) inverting β-xylosidases. The structures are of GlyA1 (PDB 5K6N; GH3; carbon atoms in pink), GsXynB1 (PDB 2BFG; GH39; green), GT2_24_00240 (PDB 4C1P; GH52; cyan), and TsXylC (PDB 3VSU; GH120; blue), which are retaining β-xylosidases, and GsXynB3 (PDB 2EXJ; GH43; white), which is an inverting β-xylosidase (see caption of Figure 3 for further details of the enzymes). Important distances (in Å) are shown next to dashed lines.

Except for the enzymes from family GH43, all β-xylosidases in the CAZy database are predicted to have a retaining mechanism (Table 1). Among these retaining β-xylosidases, structural data with bound ligand are available for enzymes from GH3 [58], GH39 [40,41,42], GH52 [73], and GH120 [25]. A structural alignment of these β-xylosidases on the basis of their bound ligand revealed that the carboxylate group of their catalytic nucleophiles, which are Glu in GH39 and GH52, and Asp in GH3 and GH120, are spatially conserved relative to the bound ligand (Figure 6a). They are within good distance (~3.1 Å) and right position for reaction with the anomeric carbon of the scissile glycosidic bond. On the other hand, the carboxylate group of their catalytic acid/base, which is Glu in GH3, GH39, and GH120, and Asp in GH52, are spatially less conserved, although they are at productive hydrogen-bonding positions (~3.2 Å on average) to the corresponding glycosidic oxygen atom.

β-Xylosidases from GH43 are inverting enzymes [79]. They use Asp and Glu as the general base and general acid, respectively [70,80,81]. Similar to the catalytic acid/base of the retaining β-xylosidases, their catalytic acid is within hydrogen-bonding distance (~2.7 Å) to the glycosidic oxygen atom of the scissile bond (Figure 6b). However, compared to the catalytic nucleophile of the retaining β-xylosidases, their catalytic base is located further away from the anomeric carbon atom of the scissile glycosidic bond with a distance of ~5.2 Å [65,66,70]. This distance provides sufficient space for accommodating a catalytic water molecule that can be activated by the catalytic base to attack the anomeric carbon [66]. It has been observed generally for GHs that the distance between the carboxylate groups of the catalytic base and acid of retaining enzymes is shorter (~5 Å) than the distance between the carboxylates of the catalytic nucleophile and acid/base of inverting enzymes (~8–10 Å) [29,82]. This is also the case for the GH43 β-xylosidases. Indeed, in the inverting GH43 β-xylosidase from *G. stearothermophilus* T-6, for example, a distance of ~7.9 Å between the carboxylate groups of its catalytic residues has been observed [66].

## 5. Inhibition of β-Xylosidases by Monosaccharides

### 5.1. Inhibition by d-xylose

Many β-xylosidases are inhibited to varying degrees by their main product d-xylose (Table 2). For example, the β-xylosidase from the fungus *Trichoderma harzianum* C is very sensitive to d-xylose inhibition; its activity is completely inhibited by the presence of only 2 mM d-xylose [83]. In contrast, the GH39 β-xylosidase from the extreme thermophilic bacterium *Dictyoglomus thermophilum* DSM 3960 is very resistant to d-xylose, with only 40% inhibition in the presence of 3 M of the sugar [84].

Most characterized β-xylosidases have significant affinity for d-xylose, with reported inhibition constants (*K*_i_) of less than 10 mM (Table 3). To our knowledge, the β-xylosidase from *Talaromyces emersonii* has the highest affinity for d-xylose with a *K*_i_ value as low as 1.3 mM [112], suggesting that the enzyme is very sensitive to inhibition by this monosaccharide. Other β-xylosidases are much less prone to d-xylose inhibition, such as those from *B. pumilus* 12 [113], *G. thermoleovorans* IT-08 [114], and uncultured bacterium [115] with *K*_i_’s of 26.2, 76, and 145 mM, respectively. So far, the β-xylosidase from the bacterium *Cellulomonas uda* [116] is the β-xylosidase with the highest reported *K*_i_ value for d-xylose, i.e., 650 mM, with the caveat that this *K*_i_ was determined using crude enzyme. Thus, in general, and as summarized in Table 3, β-xylosidases have relatively high affinity (low *K*_i_) for d-xylose and, therefore, they are susceptible to product inhibition by this sugar.

From Table 2 and Table 3 it appears that no direct relationship exists between the inhibition of β-xylosidases by d-xylose and their organismal origin or GH family. Many β-xylosidases from different bacteria and fungi suffer from such product inhibition. Likewise, product inhibition is observed for β-xylosidases belonging to different GH families. This is reasonable because all β-xylosidases bind the same substrate (d-xylose oligomers), necessitating affinity for xylosyl residues and some commonality in their active site structure (see above).

Interestingly, the activity of several β-xylosidases is stimulated by d-xylose, particularly at low concentration. The β-xylosidase from *Thermotoga thermarum* DSM 5069 was stimulated by d-xylose concentrations of up to 500 mM; its maximum activity was observed in the presence of 200 mM d-xylose, which was ~20% higher than in the absence of the sugar [96]. A similar stimulatory effect has been reported for the bifunctional β-xylosidase/α-l-arabinofuranosidase from *Phanerochaete chrysosporium* BKM-F-1767 [43] and the β-xylosidase from *Dictyoglomus thermophilum* DSM 3960 [84,158]. However, the mechanism of such stimulation is currently not known.

### 5.2. Inhibition by l-arabinose

l-arabinose is one of the monosaccharides produced during enzymatic saccharification of cellulosic biomass [7,8,28]. It is liberated by the action of α-l-arabinofuranosidases, and, during the saccharification process, its concentration may sufficiently increase to inhibit the activity of hemicellulolytic enzymes [115]. Indeed, l-arabinose has been identified as an inhibitor of various β-xylosidases (Table 4). For instance, 50 mM l-arabinose reduces the activity of the aryl β-xylosidase from *Caldocellum saccharolyticum* Tp8T6.3.3.1 by 15% [120], the β-xylosidase from *B. pumilus* 12 by 21% [113], and the bifunctional β-xylosidase/α-l-arabinofuranosidase from *P. chrysosporium* BKM-F-1767 by ~70% [43]. This is not surprising, since the stereochemistry of l-arabinose near the glycosidic bond is similar to that of d-xylose [61], explaining its binding in the active site of a β-xylosidase.

In general, l-arabinose is a weaker inhibitor of β-xylosidase activity than d-xylose. For example, the β-xylosidase from the fungus *Trichoderma reesei* RUT C30 is strongly inhibited by d-xylose with a *K*_i_ of 2.4 mM, but it is not inhibited by l-arabinose, even at a concentration of 500 mM [24]. Several other β-xylosidases can also withstand high concentrations of l-arabinose [95,122,141]. Finally, the β-xylosidase from *Enterobacter* sp. is competitively inhibited by l-arabinose, but with a quite high *K*_i_ value of 102 mM [72], indicating that the enzyme has only low affinity for l-arabinose.

Intriguingly, at low concentration (~5 mM) l-arabinose stimulates rather than inhibits the β-xylosidase activity of the bifunctional β-xylosidase/α-l-arabinofuranosidase from *P. chrysosporium* BKM-F-1767, as also noticed for d-glucose [43]. Activation by l-arabinose has also been observed for a furan aldehyde-tolerant β-xylosidase/α-l-arabinofuranosidase procured from a metagenomic sample, which showed 65% higher β-xylosidase activity compared with the control without l-arabinose [109]. Although there are many data describing the effects of l-arabinose on inhibition/activation of β-xylosidase activity, the molecular basis of the effects on activity still needs further investigation.

### 5.3. Inhibition by Other Monosaccharides

Apart from d-xylose and l-arabinose, d-glucose is another monosaccharide that has been frequently reported to affect β-xylosidase activity. d-glucose inhibits the β-xylosidase activity of the β-xylosidases from *S. ruminantium* GA192 (*K*_i_ 44 mM) [27], *B. pumilus* 12 (9% inhibition at 50 mM) [113], *T. harzianum* (3% inhibition at 5 mM) [83], and the bifunctional β-glucosidase/β-xylosidases RuBG3A and RuBG3B from the metagenome of yak rumen microorganisms (97.5% and 45.6% inhibition, respectively, at 5 mM) [111]. On the other hand, the sugar did not inhibit β-xylosidases from *Thermomonospora fusca* [159], *A. niger* 90196 [134], *A. oryzae* [138], and *N. crassa* ST A [147] at concentrations of up to 90, 20, 20, and 10 mM, respectively. The Mg^2+^-activated β-xylosidase RS223-BX could even withstand much higher d-glucose concentrations displaying a *Ki* value of 1270 mM on the substrate *p*-nitrophenyl-α-l-arabinofuranoside (*p*NPA) [19]. Apparently, inhibition by d-glucose varies considerably among β-xylosidases.

Finally, besides d-xylose, l-arabinose, and d-glucose, also other monosaccharides have been reported to inhibit β-xylosidases, including -d-arabinose [26,113], -d--erythrose [26], -d-fructose [111,138], d-galactose [26,113,120], -d-ribose, and -l-xylose [26,113]. Again, the molecular details of the interactions of these sugars with the enzymes are not known.

### 5.4. Inhibition Kinetics

The inhibition of β-xylosidases by monosaccharides follows competitive, non-competitive, or un-competitive inhibition kinetics. For most β-xylosidases, d-xylose acts as a competitive inhibitor when using *p*-nitrophenyl-β-d-xyloside (*p*NPX) as substrate [113,114,120,134]. However, the β-xylosidase from *N. crassa* ST A showed non-competitive inhibition by this sugar [147]. Furthermore, d-xylose inhibition of the β-xylosidase from *S. ruminantium* GA192, which also displays α-l-arabinofuranosidase activity, followed non-competitive kinetics for its β-xylosidase activity on *p*NPX as substrate, but competitive kinetics for its α-l-arabinofuranosidase activity on *p*NPA as substrate [26]. This differs slightly from *A. carbonarius* KLU-93 β-xylosidase, for which d-xylose was a competitive inhibitor for the conversion of both substrates [130]. Similarly, l-arabinose acts as a competitive inhibitor for the hydrolysis of *p*NPA by the RS223-BX β-xylosidase [19], but it is a non-competitive inhibitor of *S. ruminantium* GA192 β-xylosidase hydrolyzing *p*NPX or *p*NPA [26]. With respect to these substrates, -d-arabinose, d-glucose, and -d-ribose are competitive inhibitors of *S. ruminantium* GA192 β-xylosidase, whereas -d--erythrose and -l-xylose are non-competitive [26]. As also observed for *S. ruminantium* GA192 β-xylosidase, d-glucose inhibition of RS223-BX was competitive when using *p*NPA as substrate [19]. Uniquely, un-competitive inhibition was displayed by -d-fructose for the activity of *A. oryzae* β-xylosidase on *p*NPX [138]. Thus, commonly, competitive inhibition by d-xylose is observed. Other monosaccharides can display both competitive and non-competitive inhibition, and in one case, -d-fructose, un-competitive inhibition takes place. The exact mechanism and the structural details of non-competitive and un-competitive inhibition remain unknown.

### 5.5. Structural Details of Inhibitor Binding in the Active Site of β-Xylosidases

As discussed above, the active site pockets of β-xylosidases contain two substrate-binding subsites, subsites –1 and +1, on either side of the scissile bond. In the active site of *S. ruminantium* GA192 β-xylosidase, the monosaccharides -d-arabinose, l-arabinose, -d--erythrose, and -d-ribose can bind in both subsites –1 and +1, but d-xylose and -l-xylose bind only in subsite –1 [26]. Similarly, d-glucose binds in one subsite only. Its binding position was speculated to be in subsite –1, with partial occupancy of subsite +1, because glucose is too large to fit in subsite –1 only. Alternatively, it could bind in such a way that it excludes binding of a second sugar [26].

In contrast, *G. thermoleovorans* IT-08 β-xylosidase shows rather dissimilar binding properties for l-arabinose and d-xylose compared to *S. ruminantium* GA192 β-xylosidase. Crystal structures of *G. thermoleovorans* IT-08 β-xylosidase revealed that l-arabinose binds exclusively in subsite –1, while d-xylose prefers subsite +1 [70]. Thus, depending on the enzyme, the –1 and +1 subsites differ in preference for different monosaccharides, which could also contribute to the differences in inhibition kinetics observed for the different enzymes.

### 5.6. Engineering to Reduce β-Xylosidase Inhibition by Monosaccharides

During saccharification of cellulosic biomass, monosaccharides such as d-xylose, l-arabinose, and d-glucose, may reach concentrations that are high enough to inhibit β-xylosidase activity [26,27]. Therefore, β-xylosidases that are not affected by high monosaccharide concentrations are highly desirable for the efficiency of the saccharification process. To develop such β-xylosidase variants, the W145G mutation was introduced into *S. ruminantium* GA192 β-xylosidase, resulting in a variant with a 3-fold lower affinity for d-xylose and a 2-fold lower affinity for d-glucose [121]. Subjecting this variant to saturation mutagenesis of residue 145 yielded variants with even lower affinity for monosaccharides and higher catalytic activity than wild-type enzyme [27]. Mutation of Trp-145 alters the affinity of subsite +1 for d-xylose, but not that of subsite -1, where catalysis occurs, suggesting a strategy for reducing inhibition by monosaccharides by mutating residues of subsite +1 [27]. In the structure of *G. thermoleovorans* IT-08 β-xylosidase, d-xylose binds in subsite +1 interacting, among others, with Asp-198, which is not present in most other β-xylosidases [70]. Therefore, this residue may be a good target for mutation to obtain *G. thermoleovorans* IT-08 β-xylosidase variants with lower affinity for d-xylose.

## 6. Concluding Remarks

β-Xylosidases are highly diverse in their amino acid sequence. Currently, enzymes with β-xylosidase activity can be found in 11 different glycoside hydrolase families in the CAZy database, i.e., in GH families 1, 3, 5, 30, 39, 43, 51, 52, 54, 116, and 120. They fold into several distinct three-dimensional structures. While the enzymes from GH families 1, 3, 5, 30, 39, and 51 all show a (β/α)_8_ TIM-barrel structure, those from families 43, 52 and 116, 54, and 120 have as their main structural feature a 5-bladed β-propeller, a (α/α)_6_-barrel, a β-sandwich, and a right-handed parallel β-helix, respectively. Likewise, the catalytic mechanism of β-xylosidases is also varied. Although generally β-xylosidases hydrolyze their substrates with retention of the substrate’s anomeric carbon configuration, the enzymes from GH43 invert the anomeric configuration. However, despite their diversity in overall folds, all structurally characterized β-xylosidases have a typical pocket-shaped active site with two carbohydrate-binding subsites, which bind a xylobiosyl moiety of the xylooligosaccharide substrates. Unfortunately, the active sites of many β-xylosidases also possess a relatively high affinity for monosaccharides, such as xylose, arabinose, and erythrose, that competitively inhibit the enzymes’ activity. Moreover, some β-xylosidases are also non-competitively or un-competitively inhibited by monosaccharides. Such product inhibition limits the application of β-xylosidases in xylan saccharification, since high monosaccharide concentrations may easily be generated during the process. Therefore, β-xylosidases with low monosaccharide affinity are highly desirable for various applications. A random mutagenesis approach has already shown success in reducing the affinity of a β-xylosidase for d-xylose. Furthermore, with 3D structures available for a variety of β-xylosidases, rational site-directed mutagenesis may also be a good approach to render the enzymes less prone to product inhibition.

## Figures and Tables

**Table 1:** Distribution of the current β-xylosidases in the CAZy database, their catalytic domain fold, their type of catalytic mechanism, and their catalytic residues.

Family (GH)	Total Number of β-xylosidase Sequences	Clan	Overall Fold of the Catalytic Domain	Catalytic Mechanism ^†^	Nucleophile	General Acid/Base
^‡^ 1	2	A	(β/α)_8_ TIM-barrel	Retention	Glu	Glu
3	103	n.a. ^#^	(β/α)_8_ TIM-barrel	Retention	Asp	Glu
5	1	A	(β/α)_8_ TIM-barrel	Retention	Glu	Glu
30	4	A	(β/α)_8_ TIM-barrel	Retention	Glu	Glu
39	24	A	(β/α)_8_ TIM-barrel	Retention	Glu	Glu
43	96	F	5-bladed β-propeller	Inversion	Asp ^§^	Glu
51	2	A	(β/α)_8_ TIM-barrel	Retention	Glu	Glu
52	11	O	(α/α)_6_-barrel	Retention	Glu	Asp
^‡^ 54	2	n.a. ^#^	β-sandwich ^%^	Retention	Glu ^%^	Asp ^%^
^‡^ 116	1	O	(α/α)_6_-barrel	Retention	Glu	Asp
120	2	n.a. ^#^	right-handed parallel β-helix	Retention	Asp	Glu

^†^: Catalysis by GHs commonly proceeds with either retention or inversion of the substrate’s anomeric carbon configuration. See main text for further information. ^‡^: It is unknown whether the enzymes from GH1, GH54, and GH116 have β-xylosidase activity on natural substrates. ^#^: Not part of a clan. ^§^: General base; General acid. ^%^: Not assigned in the CAZy database. Data are from the crystal structure of the α-l-arabinofuranosidase from *Aspergillus kawachii* IFO4308 [38].

**Table 2:** Examples of microbial β-xylosidase inhibition by d-xylose.

Organism	GH Family	d-xylose Concentration (mM)	Inhibition (%)	Reference
**Bacteria:**				
*Bacillus halodurans* C-125	GH39	200	0	[85]
*Bacillus subtilis* M015	GH43_11	20	45	[86]
*Corynebacterium alkanolyticum* ATCC 21511	GH3	200	70	[87]
*Dictyoglomus thermophilum* DSM 3960	GH39	3000	40	[84]
*Geobacillus* sp. WSUCF1	GH39	300	50	[88]
*Geobacillus thermodenitrificans* NG80-2	GH39	400	50	[89]
*Geobacillus thermodenitrificans* NG80-2	GH43	300	50	[89]
*Geobacillus thermodenitrificans* NG80-2	GH52	600	50	[89]
*Lactobacillus brevis* ATCC 14869	GH43_11	100	20	[90]
*Lactobacillus brevis* ATCC 14869	GH43_12	100	66	[90]
*Massilia* sp. RBM26	GH43_11	500	50	[91]
*Paenibacillus woosongensis* KCTC 3953	GH43_35	100	25	[92]
*Selenomonas ruminantium* GA192	GH43_11	40	57	[93]
*Sphingobacterium* sp. HP455	GH43_1	247	50	[94]
*Thermoanaerobacterium saccharolyticum* JW/SL-YS485	GH120	200	30	[75]
*Thermotoga petrophila* DSM 13995	GH3	150	50	[95]
*Thermotoga thermarum* DSM 5069	GH3	1000	50	[96]
**Fungi:**				
*Aspergillus nidulans* CECT2544	n.a. ^#^	25	44	[97]
*Aspergillus niger* 11	n.a. ^#^	10	50	[98]
*Aspergillus niger* ADH-11	GH3	12	50	[99]
*Aureobasidium pullulans* CBS 58475	n.a. ^#^	6,6	42	[100]
*Candida utilis* IFO 0639	n.a. ^#^	300	0	[101]
*Humicola grisea* var. thermoidea	GH43_1	603	50	[102]
*Humicola insolens* Y1	GH43_1	79	50	[103]
*Humicola insolens* Y1	GH43_11	292	50	[103]
*Paecilomyces thermophila* J18	n.a. ^#^	139	50	[104]
*Phanerochaete chrysosporium* BKM-F-1767	GH43_14	50	70	[43]
*Pseudozyma hubeiensis* NCIM 3574	n.a. ^#^	75	50	[105]
*Rhizophlyctis rosea* Fischer NBRC 105426	GH43_1	100	49	[106]
*Scytalidium thermophilum* 77.7.8	n.a. ^#^	200	0	[107]
*Trichoderma harzianum* C	n.a. ^#^	2	100	[83]
*Trichoderma reesei* QM 9414	GH3	53	80	[108]
**Metagenomes:**				
Synthetic metagenome	GH43_1	20	44	[109]
Uncultured rumen metagenome	GH3	5	27	[110]
Yak rumen metagenome (RuBg3A ^§^)	GH3	5	18	[111]
Yak rumen metagenome (RuBg3B ^§^)	GH3	5	3	[111]

^#^: GH family is not assigned in the CAZy database; ^§^: Protein symbol.

**Table 3:** Examples of inhibition constants for d-xylose of β-xylosidases.

Organism	GH Family	Inhibition Constant (*K*_i_, mM)	Reference
**Bacteria:**			
*Alkaliphilus metalliredigens* QYMF	GH43_11	16.2	[18]
*Anoxybacillus* sp. 3M	GH52	21.3	[117]
*Bacillus halodurans* C-125	GH43_11	62.3	[118]
*Bacillus pumilus* 12	n.a. ^#^	26.2	[113]
*Bacillus pumilus* IPO	GH43_11	70	[18]
*Bacillus subtilis* subsp. subtilis str. 168	GH43_11	15.6	[18]
*Bacteroides ovatus* V975	GH43_1	6.6	[119]
*Caldocellum saccharolyticum* Tp8T6.3.3.1	n.a. ^#^	40.0	[120]
*Cellulomonas uda*	n.a. ^#^	650.0	[116]
*Enterobacter* sp.	GH43_11	79.9	[72]
*Geobacillus thermoleovorans* IT-08	GH43_12	76.0	[114]
*Lactobacillus brevis* ATCC 367	GH43_11	30.1	[18]
*Selenomonas ruminantium* GA192	GH43_11	6.24	[121]
*Streptomyces* sp. CH7	GH3	40.0	[122]
*Thermoanaerobacterium saccharolyticum* B6A-RI	GH39	20	[123]
*Thermobifida fusca* TM51	GH43_11	67.0	[124]
*Thermobifida halotolerans* YIM 90462^T^	GH43_11	43.8	[125]
*Thermomonospora*	n.a. ^#^	35-100	[126]
*Thermomonospora fusca* BD21	n.a. ^#^	19	[127]
**Fungi:**			
*Arxula adeninivorans* SBUG 724	n.a. ^#^	5.8	[128]
*Aspergillus awamori* X-100	GH3	7.7	[129]
*Aspergillus carbonarius* KLU-93	n.a. ^#^	1.9	[130]
*Aspergillus fumigatus*	n.a. ^#^	4.5	[131]
*Aspergillus japonicus*	GH3	2.9	[132]
*Aspergillus niger* 15	n.a. ^#^	2.9	[133]
*Aspergillus niger* 90196	GH3	8.3	[134]
*Aspergillus niger* ATCC 10864	GH3	3.3	[135]
*Aspergillus niger* NW147 (xlnD I ^§^)	GH3	9.8	[136]
*Aspergillus niger* NW147 (xlnD II ^§^)	GH3	13.2	[136]
*Aspergillus niger* van Tieghem (DSM 22593)	GH3	7.5	[137]
*Aspergillus oryzae* KBN616	GH3	2.7	[138]
*Aspergillus terreus* IJIRA 6.2	n.a. ^#^	10.5	[139]
*Aspergillus versicolor* (xylose-induced)	n.a. ^#^	5.3	[140]
*Aspergillus versicolor* (xylan-induced)	n.a. ^#^	2.0	[140]
*Aureobasidium pullulans* CBS 135684	n.a. ^#^	18.0	[141]
*Colletotrichum graminicola*	GH3	3.3	[142]
*Fusarium proliferatum* NRRL 26517	n.a. ^#^	5.0	[143]
*Fusarium verticillioides* NRRL 26518	n.a. ^#^	6.0	[144]
*Humicola insolens* Y1	GH3	29.0	[145]
*Neocallimastix frontalis* RK 21	n.a. ^#^	4.0	[146]
*Neurospora crassa* ST A (74 A)	GH3	1.7	[147]
*Penicillium janczewskii* CRM 1348	n.a. ^#^	6	[148]
*Penicillium oxalicum* 114-2	GH43	28.1	[149]
*Penicillium sclerotiorum*	n.a. ^#^	28.7	[150]
*Talaromyces amestolkiae*	GH3	1.7	[151]
*Talaromyces emersonii*	GH3	1.3	[112]
*Thermomyces lanuginosus* CAU44	GH43_1	63.0	[152]
*Trichoderma koningii* G-39	n.a. ^#^	5.0	[153]
*Trichoderma reesei* (βXTR ^§^)	GH3	2.4	[112]
*Trichoderma reesei*	GH3	1.4	[132]
*Trichoderma reesei* QM 9414	n.a. ^#^	11.0	[154]
*Trichoderma reesei* RUT C30	n.a. ^#^	2.3	[155]
*Trichoderma reesei* RUT C30	n.a. ^#^	2.4	[24]
**Plant:**			
*Saccharum officinarum* L.	n.a. ^#^	8.0	[156]
**Metagenomes:**			
Compost starter	GH43	145.0	[115]
Mixed microorganism (RS223-BX ^§^)	GH43_1	3.4	[19]
Uncultured rumen bacterium	GH30_2	10.6	[157]
Uncultured rumen bacterium	GH43_1	76.0	[157]

^#^: GH family is not assigned in the CAZy database; ^§^: Protein symbol.

**Table 4:** Examples of microbial β-xylosidase inhibition by l-arabinose.

Organism	GH Family	l-arabinose Concentration (mM)	Inhibition (%)	Reference
**Bacteria:**				
*Bacillus pumilus* 12	n.a. ^#^	50	21	[113]
*Caldocellum saccharolyticum* Tp8T6.3.3.1	n.a. ^#^	50	15	[120]
*Corynebacterium alkanolyticum* ATCC 21511	GH3	200	40	[87]
*Lactobacillus brevis* ATCC 14869	GH43_11	100	39	[90]
*Lactobacillus brevis* ATCC 14869	GH43_12	100	38	[90]
*Paenibacillus woosongensis* KCTC 3953	GH43_35	100	40	[92]
*Selenomonas ruminantium* GA192	GH43_11	80	61	[93]
**Fungi:**				
*Aspergillus niger* 11	n.a. ^#^	25	10	[98]
*Aspergillus niger* van Tieghem (DSM 22593)	GH3	200	30	[137]
*Colletotrichum graminicola*	GH3	50	15	[142]
*Penicillium oxalicum* 114-2	GH43	20	11	[149]
*Phanerochaete chrysosporium* BKM-F-1767	GH43_14	50	70	[43]

^#^: GH family is not assigned in the CAZy database.
